# Detecting changes in tobacco product marketplace prominence using social media, advertising, sales, and web traffic data: The example of Puff Bar in the United States tobacco marketplace from 2019 to 2021

**DOI:** 10.1371/journal.pone.0311723

**Published:** 2024-12-20

**Authors:** Stephanie R. Pitts, Sarah Trigger, Dannielle E. Kelley

**Affiliations:** Office of Science, Center for Tobacco Products, U.S. Food and Drug Administration, Silver Spring, Maryland, United States of America; Ministry of Health, General Health Directorate of Raparin and University of Raparin, IRAQ

## Abstract

Puff Bar, a disposable electronic nicotine delivery system (ENDS), was the ENDS brand most commonly used by U.S. youth in 2021. We explored whether Puff Bar’s rise in marketplace prominence was detectable through advertising, retail sales, social media, and web traffic data sources. We retrospectively documented potential signals of interest in and uptake of Puff Bar in the United States using metrics based on advertising (Numerator and Comperemedia), retail sales (NielsenIQ), social media (Twitter, via Sprinklr), and web traffic (Similarweb) data from January 2019 to June 2022. We selected metrics based on (1) data availability, (2) potential to graph metric longitudinally, and (3) variability in metric. We graphed metrics and assessed data patterns compared to data for Vuse, a comparator product, and in the context of regulatory events significant to Puff Bar. The number of Twitter posts that contained a Puff Bar term (social media), Puff Bar product sales measured in dollars (sales), and the number of visits to the Puff Bar website (web traffic) exhibited potential for surveilling Puff Bar due to ease of calculation, comprehensibility, and responsiveness to events. Advertising tracked through Numerator and Comperemedia did not appear to capture marketing from Puff Bar’s manufacturer or drive change in marketplace prominence. This study demonstrates how quantitative changes in metrics developed using advertising, retail sales, social media, and web traffic data sources detected changes in Puff Bar’s marketplace prominence. We conclude that low-effort, scalable, rapid signal detection capabilities can be an important part of a multi-component tobacco surveillance program.

## Introduction

Puff Bar is a disposable electronic nicotine delivery system (ENDS) marketed under the Puff brand, which has multiple ENDS including Puff Bar, Puff Plus, and Puff Flow, all bearing the Puff brand logo [1]. In the United States (U.S.), Puff Bar was the ENDS brand most commonly used by youth in 2021 [2]. In contrast, 2019 National Youth Tobacco Survey (NYTS) data showed that the ENDS brand most commonly used by youth was JUUL, a cartridge-based non-disposable ENDS, and only 2.6% of youth reported they most frequently used disposable ENDS [3, 4]. Puff Bar rapidly gained prominence in the ENDS marketplace following FDA’s January 2020 announcement of a guidance to prioritize enforcement of unauthorized cartridge-based ENDS with flavors other than tobacco and menthol [5, 6]. FDA published the draft guidance in March 2019, communicating FDA’s focus on reducing youth tobacco use through targeted removal of ENDS most commonly used by youth [7], issued the guidance in January 2020, and issued a revised guidance in April 2020. The guidance states that FDA would begin enforcement prioritization on February 6, 2020 [8].

**Fig 1:**
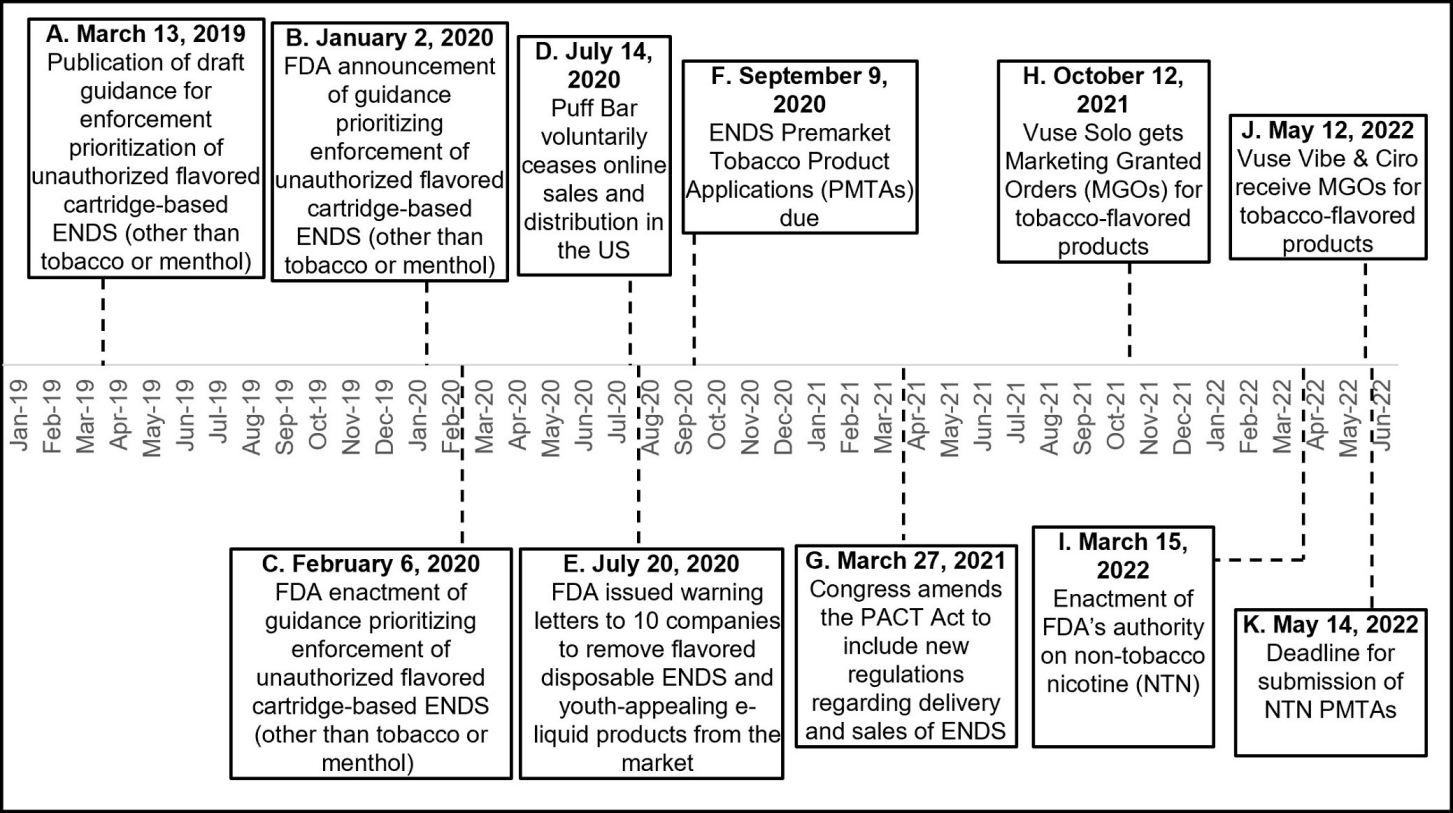
Timeline of selected key events relevant to Puff Bar marketing in the United States: January 2019 ‐ June 2022. *Note*: These selected events occurred during the study period and were relevant to Puff Bar. This is not an exhaustive list of significant tobacco regulatory and marketplace events during the study period, and does not include state, local, tribal, or territorial actions. A-K indicates the order of events and corresponds to labeling in Fig 2.

Other documented events may have also contributed to Puff Bar’s trajectory. For example, in response to increased scrutiny and youth use, Puff Bar voluntarily ceased online sales and distribution in the U.S. on July 14, 2020. On July 20, 2020, FDA issued warning letters to ten companies, including Cool Clouds Distribution, Inc. doing business as (d/b/a) Puff Bar, notifying these companies to remove their unauthorized products from the market [9, 10]. Cool Clouds Distribution, Inc. then purportedly sold the Puff Bar brand to DS Technology Licensing LLC [11]. In early 2021, Puff Bar marketed products on its website that it stated contained “tobacco-free nicotine” [12], that is, non-tobacco nicotine (NTN). Fig 1 provides a timeline of selected key events potentially associated with Puff Bar’s marketplace prominence. For purposes of this study, marketplace prominence was conceptualized as a brand’s public visibility and availability through multiple channels (public discussion, advertising, and media). This is similar to the conceptualization of perceived market prominence as “brand awareness, market share, visibility, and share-of-voice” [13], and can be thought of as a construct comprising sales, advertising, website usage, and social media discourse.

**Fig 2:**
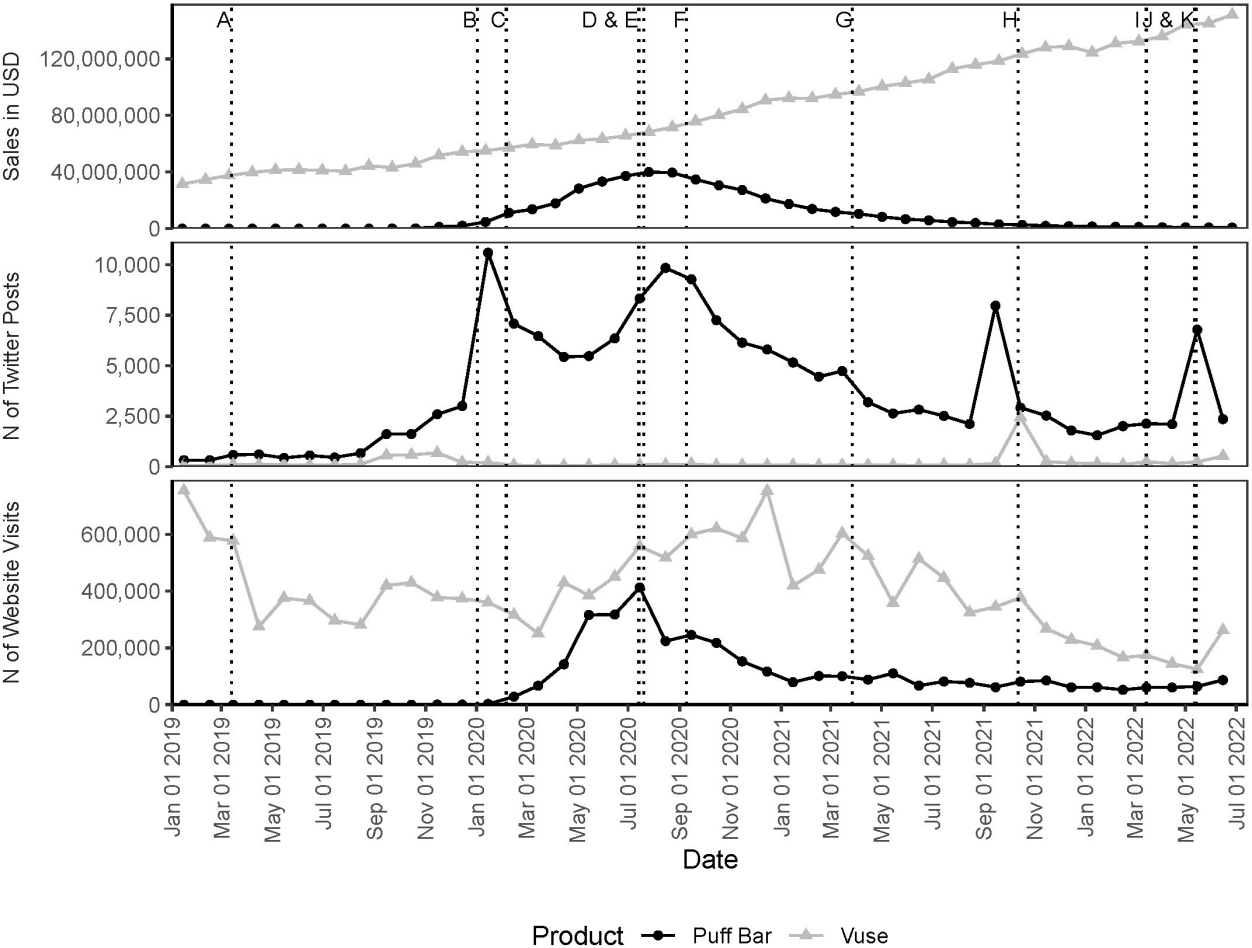
Metrics for Puff Bar and Vuse for retail sales, social media, and website traffic data for January 1, 2019–June 20, 2022. *Note*: The X axis indicates days, with tick marks for every 2 months on the 1^st^ of the month. Social media and web traffic data are aggregated by month and the data point for each month is placed on the 15th day of the month. Sales data are aggregated to 4-week periods and the data point for each 4-week period is the start date of the third week in each period. Each metric has a different y-axis representing its unit. The vertical dotted lines in the graph correspond to selected key events that may have affected Puff Bar or Vuse. Selected key events are: (A) March 13, 2019: Draft enforcement prioritization guidance released, (B) January 2, 2020: Announcement of prioritization guidance, (C) February 6, 2020: enactment of enforcement prioritization guidance, (D & E) July 14, 2020 & July 20, 2020: Puff Bar voluntarily ended online sales and FDA sends warning letters, (F) September 9, 2020: Electronic Nicotine Delivery System (ENDS) premarket tobacco product applications (PMTAs) due to FDA, (G) March 27, 2021: Preventing All Cigarette Trafficking (PACT) Act amended, (H) October 12, 2021: FDA issues marketing granted orders (MGOs) for some tobacco-flavored Vuse Solo products, (I) March 15, 2022: Enactment of FDA’s authority on non-tobacco nicotine (NTN), and (J & K) May 12, 2022 & May 14, 2022: FDA issues MGOs for some tobacco-flavored Vuse Vibe and Vuse Ciro products and the deadline for submission of NTN PMTAs to FDA.

Tobacco surveillance programs support tobacco regulatory efforts by providing data for monitoring and assessment [14, 15]. In the past few decades, tobacco surveillance programs have expanded their scope from traditional survey data [16], which describe population-level tobacco use, perceptions, exposure, and health outcomes, to include non-traditional data sources, such as advertising [17, 18], social media [19, 20], and sales data [21, 22]. Surveillance through these non-traditional sources can provide unique information on the tobacco marketplace, which contextualizes traditional survey data and may provide early signals to detect potential changes in product marketplace prominence.

To explore whether Puff Bar’s rise in marketplace prominence was detectable through non-traditional data sources (e.g., social media, advertising, web tracking, sales data), we retrospectively documented potential signals of interest in and uptake of Puff Bar using advertising, retail sales, social media, and web traffic data. For comparison, we also assessed these data for Vuse, a cartridge-based non-disposable ENDS brand. We analyzed these data sources in the context of selected key events such as FDA’s enforcement prioritization of unauthorized cartridge-based ENDS. To guide this project, we developed several research aims. Our initial aim was to determine whether social media, advertising, retail sales, and web traffic data sources capture data on Puff Bar marketplace prominence. To add context to this aim, we also addressed two follow-up aims: to identify which metrics from these data sources provide signals of potential changes in product marketplace prominence and to determine whether the metrics indicating potential changes in product marketplace prominence align with selected key events (e.g., Puff Bar’s statement of use of non-tobacco nicotine in its products). We hypothesized that changes in the selected metrics would align with some key events, such as indications of growing marketplace prominence after regulatory events which limited availability of alternative ENDS products such as unauthorized cartridge-based ENDS with flavors other than tobacco and menthol, and reduced marketplace prominence after FDA issued a warning letter to Cool Clouds Distribution, Inc. d/b/a Puff Bar.

## Methods

We gathered and analyzed data on Puff Bar from five sources to generate five study metrics (one per source). Since literature predominantly refers to “Puff Bar” when discussing any products marketed with the Puff brand logo, we used “Puff Bar” to refer to the Puff brand of products and include all Puff brand products in this analysis. We selected data sources that are available for licensing and could capture potentially relevant metrics. The five data sources included online public discourse (Twitter, via Sprinklr), sales (NielsenIQ), website visits (Similarweb), and advertising (Numerator and Comperemedia). Table 1 describes these data sources and the associated metrics. We set the study period as January 2019–June 2022 to encompass FDA’s announcement of the draft guidance on enforcement prioritization, FDA’s announcement of the final enforcement guidance on unauthorized flavored cartridge-based ENDS, the release of the 2021 NYTS data documenting youth use of Puff Bar, and Puff Bar’s marketing of ENDS purported to contain non-tobacco nicotine.

**Table 1:** Data sources used to develop brand metrics.

	Comperemedia	Numerator*	Similarweb	Sprinklr	NielsenIQ
**Type of Data Source**	Advertising	Advertising	Website analytics	Social media	Retail sales scanner
**Examples of Data Available**	Direct mail, Email, Digital display and video, Social Media, Print	Newspaper, TV networks, radio, magazines, internet displays, and outdoor	First party analytics of websites, online publicly available data, and web traffic data sources.	Publicly available information from social and web: Twitter, Reddit, Facebook, Instagram, YouTube, Forums, Quora, WordPress, print, radio, TV, Flickr, reviews, news, videos, blogs, Tumblr, classifieds, podcasts, VK (VKontakte)	Estimated dollar and unit sales based on electronic point-of-sale data from stores through product barcode checkout scanners at registers, coding of retail circulars (e.g., in-store flyers, ads promoting products), and in-store data collection (i.e., field auditors who capture in-store display information promoting products).
**Lag time for Data Availability**	One day to one month, depends on metric	None/minimal–real-time data	One month	None–real-time data	Received 7–14 days after each four-week period
**Smallest unit of time for which data can be aggregated**	Daily or monthly, depending on metric	Daily	Daily, weekly, or monthly, depending on the metric	Daily	Data aggregated to one-week increments
**Metric analyzed in this study**	Number of campaigns by month (not included due to lack of data)	Number of ads by month (not included due to lack of data)	Number of visits to brand website by month	Number of Twitter posts mentioning brand by month	Amount of sales in U.S. dollars by 4-week period

*Note*: Data availability, lag time, and smallest unit of data are based on The U.S. Food and Drug Administration Center for Tobacco Products’ (FDA/CTP’s) contracts.

* At the time this study was completed, CTP had a contract with Numerator. In 2021, Kantar acquired Numerator (https://www.numerator.com/press/kantar-acquisition-numerator-completes-creating-global-leader-shopper-behaviour-kantar/). In 2023, Vivvix launched as a combination of Kantar Media and Numerator media coverage and advertising data (https://www.vivvix.com/vivvix-launches).

We also gathered data on Vuse, a non-disposable ENDS brand owned by RJ Reynolds Vapor Company LLC, to serve as a comparison brand. We selected Vuse because it has consistently been one of the five ENDS brands with the highest number of sales in recent years [23] and may have had less media attention compared to other brands (e.g., JUUL [24]). We juxtaposed Vuse and Puff Bar data to evaluate whether Vuse and Puff Bar were experiencing concordant changes.

### Data extraction

To gather data, we searched the sources using source-specific queries related to Puff Bar and Vuse. While we use “Puff Bar” to refer to the overarching brand, additional Puff products (e.g., Puff Plus) marketed with the Puff brand logo were also included in queries. Specific queries and search methods are available in S1 File and brief query descriptions are available in Table 2. For example, on Similarweb, we queried the total number of visits by month to vusevapor.com and puffbar.com, including subdomains, from mobile and desktop devices in the U.S.

**Table 2:** Brief query descriptions by data source.

Source	Puff Bar Query Terms	Vuse Query Terms
NielsenIQ	• Brand names (e.g., Puff Bar, Puff Max, and Puff Plus) • Manufacturer name (i.e., DS Technology)	• Brand name Vuse • Manufacturer as BAT (British American Tobacco) or RJR (R. J. Reynolds Tobacco Company)
Sprinklr*	(“puff bar” OR “puffbar” OR “puff nano” OR “puff plus” OR “puffplus” OR “puff XXL” OR puff OR #puffbar OR #puffdisposable OR #puffxxl OR #puffkrush OR #puffcrush) NOT ("puff on" OR "puff activated" OR #weedlife)	“vuse alto” OR “vuse ciro” OR “vuse vibe” OR “vuse solo” OR (vuse NEAR/10 (RJR OR Reynolds OR alto OR ciro OR vibe OR solo OR ends OR ecig* OR “e-cig*” OR “e cig” OR "e cigarette" OR electronic OR vape* OR vaping OR juul*))
Numerator	“Puff” and “Puff Bar”	“Vuse”
Similarweb	puffbar.com	vusevapor.com

*Note*: *For inclusion, Tweets must have met criteria for an ENDS topic query in addition to the Puff Bar and Vuse queries.

Comperemedia, Numerator, Similarweb, and Sprinklr offer web applications that facilitate query-based searches and allow for extraction of data through download of comma-separated value (CSV) files or similar formats. NielsenIQ data are provided from the vendor in text files. Data extraction from web applications facilitated data analysis by allowing us to visualize multiple data sources in one figure. Data are not publicly available; underlying data from the results presented in the study are available through licenses from Comperemedia, NielsenIQ, Similarweb, and Sprinklr. FDA/CTP obtained access to these data through contractual agreements and the data collection and analysis methodologies complied with the terms and conditions for all data sources used in this study.

### Data analysis

We generated metrics using the data from the sources and selected these metrics based on (1) data availability for Puff Bar and Vuse, (2) potential to graph the metric longitudinally, including the time-period and unit of data provided (e.g., number of posts by day, dollar amount of sales by week), and (3) variability in metric. We graphed the metrics in order to (1) visually inspect data patterns that could serve as signals, (2) examine data patterns alongside the timeline of selected key events and metrics created with Vuse brand data, and (3) identify benefits and limitations of each data source. Given the nature of the data sources and our research aims, we determined that visualizing and describing quantitative metrics was the appropriate methodological approach for this work [21, 25–27].

We completed a statement of self-determination of exemption status for Institutional Review Board review for this study in accordance with the FDA/CTP Research Involving Human Subjects Committee policy.

## Results

### Assessing the metrics

We found Puff Bar data in social media, sales, and web traffic data. We calculated metrics for the number of Twitter posts that contained a Puff Bar key term (social media), sales of Puff Bar products measured in dollars (sales), and the number of visits to the Puff Bar website (web traffic) for January 2019–June 2022; Fig 2 presents these metrics along with corresponding metrics for Vuse. The time periods for which data were aggregated for social media, web traffic, and sales data were incongruent. In order to visualize these data together, we created monthly metrics for social media and web traffic data and 4-week time period metrics for sales data. We checked the Twitter query for robustness and found that when “puff” as a standalone search term was removed from the query for Puff Bar, the number of Puff Bar-related Twitter posts for the entire study period decreased by 17.4%, a reduction of 25,333 posts from the 145,367 total posts.

Advertising tracked through Numerator and Comperemedia during the study period captured a very limited number of Puff Bar advertisements (ads). In Numerator data, four ads containing Puff Bar products appeared from July 13, 2020–January 17, 2022. Only one of these ads was placed by Puff Bar’s manufacturer and was an online display ad that first ran on January 17, 2022. Three ads were from third-party advertisers (e.g., Vapor4Life, Volcano E-cigs) featuring Puff Bar and appeared from July 13, 2020–August 25, 2021, prior to the single Puff Bar placed ad. We did not find any data on ads for Puff Bar from Comperemedia. We did not include a metric for advertising data in Fig 2 because advertising data on Puff Bar were scant and did not allow creation of a metric that could be graphed longitudinally.

For Puff Bar, Twitter, sales, and web traffic data increased starting in late 2019 or early 2020, peaked in mid-2020, and decreased from late 2020 into 2021.

### Comparing Puff Bar data and selected key events

Puff Bar metrics for sales, web traffic, and social media all appeared to show some concordance with selected key events, as seen in Fig 2. In January and February 2020 when the enforcement guidance was announced and enacted, respectively, Puff Bar sales and web traffic increased slightly and a large spike in Twitter posts occurred. Then, in July 2020, when Puff Bar voluntarily ended online sales and FDA sent warning letters to 10 companies [9] (including Puff Bar’s manufacturer), Puff Bar website visits declined sharply, sales peaked and began to decline, and Twitter posts increased slightly for a month followed by a decline. Puff Bar-related Twitter posts spiked in May 2022, when FDA issued MGOs for some tobacco-flavored Vuse products and FDA’s May 14, 2022 deadline for companies to submit premarket tobacco product applications (PMTAs) for non-tobacco nicotine products on the market as of April 14, 2022 [28] occurred. However, internal analysis suggested the increase in Twitter posts may have been driven by a viral post about a celebrity using Puff Bar. No other events appeared to be concordant with observed changes in metric values within the study period, and some observed changes appear unrelated to events.

#### Comparing patterns in Puff Bar and Vuse data

We compared metrics for Puff Bar and Vuse (Fig 2). Sales and website visits were higher for Vuse than for Puff Bar throughout the entire study period. Vuse sales grew steadily while sales of Puff Bar peaked in mid-2020 and subsequently declined through the end of the study period. Website visits for vusevapor.com were volatile, showing increases and decreases that do not appear to be concordant with the key events included in Fig 1 (e.g., for 2021, many points of inflection in Vuse website visits did not appear to be concordant with events, as seen in Fig 2). However, an increase in visits to vusevapor.com was concordant with the increase in visits to puffbar.com and continued past August 2020, when visits to puffbar.com began to decrease. From 2021 through the end of the study period, visits to vusevapor.com generally decreased, with a small spike in visits for June 2022, the last month of the study. The number of Twitter posts mentioning Puff Bar was higher than the number mentioning Vuse. Twitter posts for Vuse remained low and consistent across the study period, with the exception of October 2021, when FDA issued marketing granted orders (MGOs) for some tobacco-flavored Vuse products [29]. However, there was a small increase in Twitter posts about Vuse from September-November 2019 (roughly the same timeframe when Twitter posts about Puff Bar began to increase and manufacturers may have been anticipating the previously announced enforcement prioritization) followed by a drop in December 2019–January 2020 back to baseline, when the metric for Puff Bar spiked.

In contrast to the lack of advertising data for Puff Bar, Vuse advertising data were available for the study period. For example, Comperemedia’s database included 71 (2019) and 214 (2020) Vuse e-mail and mail campaigns.

## Discussion

Surveillance programs can use publicly available data, including social media and web traffic data, and data sources available for licensing such as sales data, to capture rapid changes in the U.S. tobacco marketplace. We found that analyzing data to create metrics for each of these non-traditional data sources over a defined time-period (for example, total monthly website visits) provided signals that can detect potential changes in a tobacco product’s marketplace prominence. In support of this, we found that changes in these metrics exhibited some concordance with major selected key events, including concordance with the expected direction of changes (that is, inflection points where metrics changed from increasing to decreasing, or vice versa). However, changes in metrics did not always coincide with timing of selected key events and may be related to events that we did not capture.

The query methods used in this retrospective study required that the analysts were aware of the brands selected for surveillance. Furthermore, the query methods required manual generation of brand-specific queries, which imposes scalability limitations. Understanding the trajectory of past popular products may provide insight into prospective surveillance for new products with the potential for rapidly increasing use. Complementary research has explored brand discovery methods that can provide lists of potential brands for surveillance [30].

### Utility of multiple sources

Each data source may serve a complementary role in surveillance. For example, in our study, early detection of initial public awareness of Puff Bar could have been achieved by surveilling the number of Puff Bar-related Twitter posts, the first metric to show an increase and the only metric for which Puff Bar had a higher value compared to Vuse. Social media data may be highly responsive to new products that have sparked public attention and interest, and in the case of Puff Bar, may have served as an early indicator of Puff Bar’s change in marketplace prominence. Alternatively, spikes in social media posts about a brand could be indicative of the brand’s use of social media-based advertising, among other alternative contributing actions. Sales and website visits are indicators of individual and population-level behaviors (in the case of sales data, product purchasing and associated use behaviors, including trial of new products; in the case of website visits, information seeking and product purchase), rather than public social engagement. The metric for Twitter posts that mention Vuse exhibited an increase concordant with posts that mention Puff Bar. This could have indicated increased public discussion or tobacco manufacturer and retailer advertising of many ENDS.

Alternatively, in this study, web traffic data in particular, and sales data to a lesser extent, appear to detect a decline in Puff Bar marketplace prominence concordant with FDA’s issuance of warning letters and Puff Bar’s voluntary termination of online sales more quickly than social media data. Puff Bar’s cessation of online sales may have particularly affected website visits because people could no longer purchase products from the Puff Bar website and Puff Bar may have made other changes to its digital presence that reduced traffic to its site. Similarly, visits to vusevapor.com declined during the latter third of the study period, possibly related to legislation that reduces online ENDS sales by putting ENDS shipping restrictions in place [31]. Other possible explanations for the reduction in visits are potential changes to website accessibility of which we are not aware (such as requiring login to access the website) or other reasons such as public concern about youth ENDS access and subsequent reactions by ENDS manufacturers to change marketing practices, both of which have been reported elsewhere [32].

It is important to consider each metric in context. It is intuitive that website visits might immediately decrease when a manufacturer ceases online sales, and indeed, we saw a steep decline in visits to Puff Bar’s website at that time. We could expect potentially different changes in patterns of Twitter postings and sales in response to the manufacturer’s action to stop selling Puff Bar online and FDA’s warning letters. Twitter posts might initially increase as people share news articles and their opinions on Puff Bar and regulatory actions on Twitter. NielsenIQ sales data might be unresponsive to the termination of online Puff Bar sales because online sales are not captured in these data. Retailer sales may decline slowly if retailers sell the remainder of their inventories.

Examining trends and patterns in metrics from non-traditional sources is useful for ongoing surveillance, including searching for and detecting potential changes in the tobacco marketplace. However, these data do not replace product use data collected through surveys (e.g., the National Youth Tobacco Survey [33]), cohort studies (e.g., the Population Assessment for Tobacco and Health Study [16]), and other more traditional methods. Changes in metrics from non-traditional data sources can serve as signals for deeper investigation or provide information to inform future research, including creating or revising survey items for population surveys. Alone, each metric from one of these non-traditional data sources may be able to rapidly detect a potential change in the tobacco marketplace and a signal from a metric may warrant further investigation. Each source captures unique activity, and triangulation across the sources may provide stronger evidence that a marketplace change is actually occurring. Furthermore, data from non-traditional sources can be examined using automated methods [34, 35] to increase their utility for rapid surveillance.

### Noise in metrics and false signals

Metrics from social media, sales, website analytics, and advertising data may be noisier (i.e., distorted) than more traditional surveillance data, and may be variably noisy by brand. For example, there were more Twitter posts on Puff Bar compared to Vuse; however, “puff” is a generic term and could have captured more extraneous posts unrelated to the Puff Bar brand compared to the number of extraneous posts captured by the more unique term “vuse.” We found that when “puff” as a standalone search term was removed from the query for Puff Bar, the number of Puff Bar-related Twitter posts for the entire study period decreased. By including “puff,” we captured many posts that used “puff” alone to refer to Puff Bar, and the findings appears robust regardless of the inclusion or exclusion of the term. Of note, our keyword queries were run on a corpus of data that were collected using a keyword query for ENDS (S1 File), and therefore post relevance was likely higher than if we used unfiltered Twitter data. Similarly, our query for Numerator advertising data also located numerous posts unrelated to Puff Bar. Advertising data may be noisy for brands that contain moderately generic terms or have brand names that are used by multiple companies.

Interpreting changes in metrics from non-traditional data sources to understand broad tobacco marketplace changes can be challenging. For example, in advertising data, ads featuring Puff Bar generally came from retailers and appeared after Puff Bar sales had increased. Retailers may have run these ads to spur interest in their stores by capitalizing on increased sales of Puff Bar and other disposables rather than to increase sales of Puff Bar specifically. In this case, the metric likely showed sustained marketplace prominence rather than a change. Changes in visits to websites were often volatile and appeared unrelated to the selected key events but may be concordant with website-based marketing tactics such as sweepstakes or coupon deals rather than increased marketplace prominence. Furthermore, we observed some spikes in Twitter data when sensational posts mentioned Puff Bar and gained virality, but they likely were not indicative of a change in Puff Bar marketplace prominence.

### Limitations

Our study methodology is limited in its ability to extrapolate findings to inform tobacco marketplace surveillance. We only included one metric per data source in our analysis; however, the data sources provided multiple metrics and access to raw data, which can be analyzed and transformed to augment our metrics’ utility. Data transformations may help to dampen noise and highlight changes in the metrics.

We collected data retrospectively. Data providers frequently update raw data and data processing algorithms to improve the quality of metrics. Therefore, we are unsure whether the data we used are representative of data available in real or near-real time during the study period or whether data had been edited. For some sources, data were unavailable for the full or partial study period. For example, we did not have website visit data available for puffbar.com until November 2019 and Comperemedia did not have any data available on Puff Bar. We view this both as a result and a limitation. Furthermore, some sources, like sales data sources, can take multiple months to index a new product for inclusion in data, and real-time data may have been lacking on Puff Bar early in the study period. Therefore, data used in this analysis may not reflect data available in real or near-real time for rapid surveillance. Additionally, our data only covers a set time-period. The study period selected does not include the FDA Commissioner’s November 2018 initial statement directing FDA/CTP to revise the compliance policy for flavored ENDS, excluding ENDS flavored as tobacco, mint, menthol, or non-flavored [36]. Data and events outside of our study period, including state, local, tribal, or territorial actions, may have provided useful information.

Finally, we focused our analyses only on one brand, Puff Bar, and conclusions on data availability and the utility of each data source for surveillance may not apply to other brands or tobacco products. This limited the generalizability of this study to other tobacco products and brands. While we included data from a wide variety of sources, our sources are not comprehensive and other sources with useful data may be available (e.g., point-of-sale advertising). We include study-specific limitations of each data source in S1 File.

## Conclusions

This study demonstrates how quantitative changes in metrics from non-traditional data sources may have detected changes in Puff Bar’s marketplace prominence. The tobacco marketplace is large and rapidly changing, with many products produced by different manufacturers and marketed under different brands [37]. Overall, our results suggest that multiple complementary non-traditional data sources may be operationalized for a multi-component tobacco product marketplace surveillance program. With the availability of new and varied data sources and analytical methodologies, there are many avenues in which tobacco surveillance programs can be designed and implemented. We conclude that scalable, rapid signal detection capabilities may be an important part of a multi-component tobacco surveillance program.

## Supporting information

S1 File Additional information on data sources. (DOCX)
