# Supplementary material for: Detecting changes in tobacco product marketplace prominence using social media, advertising, sales, and web traffic data: The example of Puff Bar in the United States tobacco marketplace from 2019 to 2021
Source: PLoS One. 2024 Dec 20;19(12):e0311723. doi: 10.1371/journal.pone.0311723 (PMC11661582; doi:10.1371/journal.pone.0311723)
Supplement: S1 File — (DOCX) [file pone.0311723.s001.docx]

**S1:** **Additional Information on Data Sources**

**NielsenIQ**

**Data Source**

The U.S. Food and Drug Administration Center for Tobacco Products (CTP) licenses NielsenIQ weekly in-store sales scanner data (also known as Retail Measurement Services or RMS) collected from brick-and-mortar retailers for expanded all-outlets combined (e.g., food/grocery, drug, discount/dollar, mass merchandisers, and military commissary stores) and for convenience stores.

**Methods**

We searched the NielsenIQ data for relevant brand names (e.g., Puff Bar) as well as the manufacturer name (i.e., DS Technology -- NielsenIQ updated files to list DS Technology as the manufacturer for Puff Bar instead of Cool Clouds, regardless of date). Through this process, we used combined data for the brand names Puff Bar, Puff Max, and Puff Plus, all of which had the manufacturer listed as DS Technology. For Vuse, we searched for the brand name Vuse and the manufacturer as BAT (British American Tobacco) or RJR (R. J. Reynolds Tobacco Company).

Data files were searched from December 30, 2018 to July 9, 2022. We used brand dollar sales for analysis. NielsenIQ data is provided in one-week increments; we looked at four-week periods to align with the other data sources.

**Limitations**

NielsenIQ uses proprietary methods to collect the data. Some NielsenIQ data are based on projections or extrapolations; data should be viewed as estimates and not exact counts.

The NielsenIQ data licensed by CTP do not include tobacco/nicotine data from certain outlets, including but not limited to: liquor stores; food stores with annual sales of less than $2 million (e.g., bodegas); drug stores with annual sales of less than $1 million, certain club stores (e.g., Costco); certain dollar stores (e.g., Dollar Tree); internet sales from point-of-sale retailers; and specialty tobacco stores, including vape shops, cigar bars, and hookah bars. In addition, the NielsenIQ data cover tobacco/nicotine product sales from only the 48 contiguous states, and does not include sales data from Hawaii and Alaska.

NielsenIQ states it typically takes at least 3 months to include new products in its data. The NielsenIQ data licensed by CTP do not include tobacco/nicotine data for products without a UPC code. NielsenIQ data identify attributes based only on external product packaging and labeling. Data may underrepresent sales of ENDS.

**Data Source Disclosure**

The authors’ own analyses, calculations and conclusions were informed in part by the NielsenIQ data through NielsenIQ’s Retail Measurement Service (RMS) for the Tobacco Alternatives category for the four-year annual time periods through the week ending July 9, 2022 for Total U.S. Expanded All Outlets Combined (xAOC) and convenience stores, and are those of the FDA and do not reflect the views of NielsenIQ. NielsenIQ is not responsible for, had no role in, and was not involved in analyzing and preparing the results reported herein, or in developing, reviewing, or confirming the research approaches used in connection with this report. NielsenIQ RMS data consist of weekly purchase and pricing data generated from participating retail store point-of-sale systems in all U.S. markets. See http://www.NielsenIQ.com/global/en/ for more information.

**Sprinklr**

**Data Source**

Sprinklr is a social media monitoring service licensed to track public conversations on select social media and web sources. We used Sprinklr to access public conversation on Twitter, a microblogging platform.

**Methods**

We searched Twitter through Sprinklr’s Listening Insight tool for January 01, 2019 through June 30, 2022. We searched for Puff Bar- and Vuse-related Tweets within an existing ENDS topic corpus using the Puff Bar and Vuse queries below. We limited our search to only publicly available posts made in English and originating from the U.S. or an unknown location. Sprinklr offers data from multiple social media platforms; however, we only included Twitter data because Sprinklr provides comprehensive access to Twitter data, allowing us to maintain a consistent trendline.

*Puff Bar Query*

(“puff bar” OR “puffbar” OR “puff nano” OR “puff plus” OR “puffplus” OR “puff XXL” OR puff OR #puffbar OR #puffdisposable OR #puffxxl OR #puffkrush OR #puffcrush) NOT ("puff on" OR "puff activated" OR #weedlife)

*Vuse Query*

“vuse alto” OR “vuse ciro” OR “vuse vibe” OR “vuse solo” OR (vuse NEAR/10 (RJR OR Reynolds OR alto OR ciro OR vibe OR solo OR ends OR ecig* OR “e-cig*” OR “e cig” OR "e cigarette" OR electronic OR vape* OR vaping OR juul*))

After obtaining the Twitter data, we aggregated the number of posts per month to create a metric.

**Limitations**

Not all social media platforms are tracked in Sprinklr (e.g., Snapchat, TikTok) and not all tracked platforms provide substantial posts due to privacy settings and age-gating (e.g., Facebook, Instagram, Reddit). Data collected are limited to publicly available posts and do not include private posts.

Results may not be representative of all Twitter users, all social media users, or the general population. Most Tweets are not geotagged, and we do not know if they originated from the U.S. or elsewhere. It is possible that some important keywords were omitted from the queries and therefore some relevant content may be missing. Furthermore, there may be irrelevant keywords included in the query; therefore, some content captured may be unrelated to Puff Bar or Vuse.

*Full ENDS Query*

((("electronic nicotine delivery system" OR "electronic nicotine delivery systems" OR "ENDS fda"~2 OR "ENDS device"~2 OR "ENDS devices"~2 OR "nicotine salt" OR "nicotine salts") OR (("apollo" OR "aspire" OR "blu" OR "eon" OR "ezzy" OR "glas" OR "green smoke" OR "haus" OR "helix" OR "hyde" OR "jak" OR "leap" OR "leapgo" OR "logic power" OR "markTen" OR "mark ten" OR "mistic" OR "myle" OR "mig" OR "mojo" OR "moti" OR "njoy" OR "phix" OR "oro" OR "riptide" OR "ripstick" OR "ryse" OR "stig" OR "suorin" OR "sourin" OR "air bar" OR "switch mods") NEAR/5 ("FDA" OR "tobacco" OR "nicotine" OR "vaporizer" OR "vaporizers" OR "vapor" OR "vapors" OR "pod" OR "pods" OR "cig" OR "cigs" OR "cigarette" OR "cigarettes" OR "FDATobacco")) OR ("ecig" OR "ecigs" OR "ecigarette" OR "ecigarettes" OR "e cig" OR "e cigs" OR "e cigarette" OR "e cigarettes" OR "electronic cig" OR "electronic cigs" OR "electronic cigarette" OR "electronic cigarettes" OR "electroniccig" OR "electroniccigs" OR "electroniccigarette" OR "electroniccigarettes") OR ("ejuice" OR "ejuices" OR "ejuicing" OR "e juice" OR "e juices" OR "e juicing" OR "electronic juice" OR "electronic juices" OR "electronic juicing" OR "electronicjuice" OR "electronicjuices" OR "electronicjuicing") OR ("eliquid" OR "eliquids" OR "e liquid" OR "e liquids" OR "electronic liquid" OR "electronic liquids" OR "electronicliquid" OR "electronicliquids") OR ("ehookah" OR "ehookahs" OR "e hookah" OR "e hookahs" OR "electronic hookah" OR "electronic hookahs" OR "electronichookah" OR "electronichookahs") OR ("epipe" OR "epipes" OR "e pipe" OR "e pipes" OR "electronic pipe" OR "electronic pipes" OR "electronicpipe" OR "electronicpipes") OR ("vape" OR "vapes" OR "vaped" OR "vaping") OR ("podmod" OR "podmods" OR "pod mod" OR "pod mods" OR "vape pod" OR "vape pods" OR "vapepod" OR "vapepods" OR "pod systems" OR "pod system" OR "podsystems" OR "podsystem") OR ("21st century smoke" OR "21 st century smoke" OR "4x pods" OR "aspire breeze" OR "Bidi Stick" OR "BidiStick" OR "Bidi Sticks" OR "BidiSticks" OR "myblu" OR "blvk unicorn" OR "blvkunicorn" OR "bud vape" OR "budvape" OR "bud vapes" OR "budvapes" OR "cig2o" OR "cig20" OR "cali bar" OR "eleaf" OR "eonsmoke" OR "eon smoke" OR "epicvapor" OR "epicvapors" OR "epic vapor" OR "epic vapors" OR "fin elite pod"~2 OR "fin elite pods"~2 OR "geek vape" OR "geekvape" OR "geek vapes" OR "geekvapes" OR "glasvapor" OR "greensmartliving" OR "helixbar" OR "hyppe" OR "hyppebar" OR "innokin" OR "jak epic"~2 OR "joyetech" OR "juul" OR "juuls" OR "juuling" OR "juuled" OR "kado stealth"~2 OR "kandypen" OR "kandypens" OR "candypen" OR "candy pen" OR "kandy pens" OR "candy pens" OR "kangertech" OR "kanger tech" OR "logic vapeleaf"~2 OR "logicvape" OR "logicvapes" OR "lost vape" OR "lostvape" OR "mana stick" OR "manastick" OR "mana sticks" OR "manasticks" OR "mig vapor" OR "migvapor" OR "mojovape" OR "moti piin" OR "motipiin" OR "omni vapor" OR "omnivape" OR "omnivapes" OR "omni disposable" OR "oro bar" OR "orobar" OR "pop vapor" OR "pop disposable" OR "puffbar" OR "puff bar" OR "pufbar" OR "puf bar" OR "ryse bar" OR "rysebar" OR "sigelei" OR "smok" OR "snowwolf" OR "stigvgod" OR "suorin air" OR "sourin air" OR "suorinair" OR "sourinair" OR "vaporesso" OR "vgod" OR "voopoo" OR "vuse" OR "puff plus" OR "puff xxl")) AND NOT (("enter to win" OR "enter 2 win" OR "enter and win" OR "enter you can win" OR "enter your could win" OR "enters to win" OR "enters 2 win" OR "enters and wins" OR "entering to win" OR "entering 2 win" OR "entering and winning" OR "entering you can win" OR "entering your could win" OR "chance to win" OR "chance 2 win" OR "chance and win" OR "chance can win" OR "chance could win" OR "chance you can win" OR "chance your could win" OR "chance of winning" OR "chances to win" OR "chances 2 win" OR "chances of winning" OR "follow to win" OR "follow 2 win" OR "follow and win" OR "follow can win" OR "follow could win" OR "follow you can win" OR "follow your could win" OR "share to win" OR "share 2 win" OR "share and win" OR "share can win" OR "share could win" OR "share you can win" OR "share your could win" OR "comment to win" OR "comment 2 win" OR "comment and win" OR "comment can win" OR "comment could win" OR "comment you can win" OR "comment your could win" OR "comment and you can win" OR "comment and your could win" OR "like to win" OR "like 2 win" OR "like and win" OR "like can win" OR "like could win" OR "like you can win" OR "like your could win" OR "like and you can win" OR "like and your could win" OR "Retweet to win" OR "Retweet 2 win" OR "Retweet and win" OR "Retweet can win" OR "Retweet could win" OR "Retweet you can win" OR "Retweet your could win" OR "Retweet and you can win" OR "Retweet and your could win" OR "RT to win" OR "RT 2 win" OR "RT and win" OR "RT can win" OR "RT could win" OR "RT you can win" OR "RT your could win" OR "RT and you can win" OR "RT and your could win" OR "sweepstake" OR "sweepstakes" OR "Claim your" OR "Win a brand new" OR "entertowin" OR "chancetowin" OR "#job" OR "jobs" OR "bodybuilding" OR "fitness" OR "off £"~2 OR "off $"~2 OR "off €"~2 OR "off %"~2 OR "off percent"~2 OR "blu ray" OR "blu rays" OR "#できたてころね") OR "@VGOD_Finance" OR "#VirtualGod" OR "tigray" OR "#tigray"))

**Numerator**

**Data Source**

Numerator is a market surveillance and research service that monitors ads and ad expenditures for U.S. print, TV, mobile, online, radio, outdoor, direct mail and on demand services (e.g., Hulu, Paramount+).

**Methods**

To identify ads for Puff Bar, we searched for “Puff” and “Puff Bar.” To identify Vuse ads, we searched “Vuse.” For both the Puff Bar and Vuse searches, we restricted the search to ads appearing between January 1, 2019 and June 30, 2022.

The results of each search were manually reviewed for relevance. For searches related to Puff Bar, this process was more intensive as the search produced a number of ads featuring the word “puff” as a descriptor of a product not related to Puff Bar. Ads featuring “puff” as a product description not related to Puff Bar were excluded.

**Limitations**

Numerator does not capture all advertisements in the U.S. because it harvests ads from major markets and consumer reports. Furthermore, ad classification may change over time. For instance, an ad may initially be classified as first running (i.e., first appearing) on a date within the study period but may later be categorized as first running on a date outside of the study period.

**Similarweb**

**Data Source**

Similarweb is a website analytics monitoring service that tracks internet traffic and digital advertising through first-party analytics of websites (e.g., Google Analytics), online publicly available data (webpage images), global partners including demand-side platforms and internet providers, and anonymous traffic data collected from personal devices worldwide with Similarweb products installed. Similarweb processes these data to predict values for website traffic metrics.

**Methods**

We entered the domain names for brand-official Vuse and Puffbar websites (vusevapor.com and puffbar.com) into the Competitive Research tab of the Similarweb PRO platform. We set Similarweb options to include subdomains, include data for the U.S., and include data for all devices. We excluded data from outside of the U.S. We downloaded data for the metric “total visits” for vusevapor.com and puffbar.com.

Our license allows access to the past three years of data. On our original data pull (August 13, 2021), we gathered data from January 1, 2019 to December 31, 2020. We later updated our data pull to capture the past 3 years of data, but we were unable at that point in time to capture data from January 1, 2019 to May 31, 2019. Therefore, the data for January 1, 2019-May 31, 2019 are from our original data pull executed on August 13, 2021 and the data for June 1, 2019-June 30, 2022 were pulled on August 4, 2022. Data for puffbar.com for November 2019-January 2020 show as “<5,000.00” when data are downloaded as an excel file from Similarweb’s website but are presented as integers on the website itself. We copied the integers into the excel file from Similarweb in order to graph the data.

**Limitations**

Data are estimates and may be updated as Similarweb refines its data acquisition, processing, and estimation methodologies. Data for some metrics for some websites are unavailable from Similarweb due to low website traffic and inability to generate estimates.

**Comperemedia**

**Data Source**

Comperemedia is an advertising monitoring service that tracks advertisement creatives and estimated media spend for direct mail, email, digital display and video, social media, and print.

**Methods**

We searched both Comperemedia Direct and Omni for data related to Puff Bar and Vuse. Omni indexes data by company/manufacturer name and can be searched by typing in potentially relevant company names. To locate Puff Bar-related data, we typed in “Puff Bar,” “Puffbar,” “Puff,” “Cool Clouds,” “Cool Cloud,” “DS Technology,” “DS Tech,” “D.S. Technology,” “DS Technology Licensing, LLC,” “EVO Brands,” and “EVO Brand.” This list includes a number of potential spellings for thoroughness. We did not locate any companies or manufacturers related to Puff Bar in the Omni database. We located data for Vuse in Direct under the company name “R.J. Reynolds Vapor Company.” In Direct, we searched for Puff Bar-related key words using the query: “Puff” OR “Cool Clouds” OR “DS Technologies” OR “DS Technology” OR “EVO Brands” OR “EVO Brand”, with search parameters set only to the tobacco domain and the January 01, 2019-June 30, 2022 timeframe. We did not locate any Puff Bar-related data in Direct. We searched Direct using these same search parameters with the key word “Vuse” to locate data for Vuse, and found data indexed under multiple company names including “R.J. Reynolds Vapor Company,” “RJR VAPOR CO, LLC,” and “RJ Reynolds Tobacco Company.”

**Limitations**

Comperemedia does not index all companies in Omni, and it is unclear what criteria Comperemedia uses to determine whether a company should be indexed. Direct can be searched using keywords; however, when we used common keywords such as “Puff,” we located content that was unrelated to Puff Bar. Comperemedia does not offer data on some traditional advertising channels such as television or radio or include business-to-business communications.
